# An Epigenetically Distinct Subset of Children With Autism Spectrum Disorder Resulting From Differences in Blood Cell Composition

**DOI:** 10.3389/fneur.2021.612817

**Published:** 2021-04-16

**Authors:** Maryam Jangjoo, Sarah J. Goodman, Sanaa Choufani, Brett Trost, Stephen W. Scherer, Elizabeth Kelley, Muhammad Ayub, Rob Nicolson, Stelios Georgiades, Jennifer Crosbie, Russell Schachar, Evdokia Anagnostou, Eyal Grunebaum, Rosanna Weksberg

**Affiliations:** ^1^Genetics and Genome Biology, The Hospital for Sick Children, Toronto, ON, Canada; ^2^The Centre for Applied Genomics, The Hospital for Sick Children, Toronto, ON, Canada; ^3^Department of Molecular Genetics, University of Toronto, Toronto, ON, Canada; ^4^McLaughlin Centre, University of Toronto, Toronto, ON, Canada; ^5^Department of Psychiatry, Queen's University, Kingston, ON, Canada; ^6^Department of Psychiatry, University of Western Ontario, London, ON, Canada; ^7^Department of Psychiatry and Behavioural Neurosciences, Offord Centre for Child Studies, McMaster University, Hamilton, ON, Canada; ^8^Neurosciences and Mental Health Program, The Hospital for Sick Children, Toronto, ON, Canada; ^9^Department of Psychiatry, University of Toronto, Toronto, ON, Canada; ^10^Institute of Medical Science, School of Graduate Studies, University of Toronto, Toronto, ON, Canada; ^11^Holland Bloorview Kids Rehabilitation Hospital, Toronto, ON, Canada; ^12^Department of Pediatrics, University of Toronto, Toronto, ON, Canada; ^13^Division of Immunology and Allergy, The Hospital for Sick Children, Toronto, ON, Canada; ^14^Developmental and Stem Cell Biology Program, The Hospital for Sick Children, Toronto, ON, Canada; ^15^Division of Clinical and Metabolic Genetics, The Hospital for Sick Children, Toronto, ON, Canada

**Keywords:** ASD, DNA methylation, epigenetics, granulocytes, blood cell proportion

## Abstract

**Background:** Autism spectrum disorder (ASD) is a complex neurodevelopmental disorder that often involves impaired cognition, communication difficulties and restrictive, repetitive behaviors. ASD is extremely heterogeneous both clinically and etiologically, which represents one of the greatest challenges in studying the molecular underpinnings of ASD. While hundreds of ASD-associated genes have been identified that confer varying degrees of risk, no single gene variant accounts for >1% of ASD cases. Notably, a large number of ASD-risk genes function as epigenetic regulators, indicating potential epigenetic dysregulation in ASD. As such, we compared genome-wide DNA methylation (DNAm) in the blood of children with ASD (*n* = 265) to samples from age- and sex-matched, neurotypical controls (*n* = 122) using the Illumina Infinium HumanMethylation450 arrays.

**Results:** While DNAm patterns did not distinctly separate ASD cases from controls, our analysis identified an epigenetically unique subset of ASD cases (*n* = 32); these individuals exhibited significant differential methylation from both controls than the remaining ASD cases. The CpG sites at which this subset was differentially methylated mapped to known ASD risk genes that encode proteins of the nervous and immune systems. Moreover, the observed DNAm differences were attributable to altered blood cell composition, i.e., lower granulocyte proportion and granulocyte-to-lymphocyte ratio in the ASD subset, as compared to the remaining ASD cases and controls. This ASD subset did not differ from the rest of the ASD cases in the frequency or type of high-risk genomic variants.

**Conclusion:** Within our ASD cohort, we identified a subset of individuals that exhibit differential methylation from both controls and the remaining ASD group tightly associated with shifts in immune cell type proportions. This is an important feature that should be assessed in all epigenetic studies of blood cells in ASD. This finding also builds on past reports of changes in the immune systems of children with ASD, supporting the potential role of altered immunological mechanisms in the complex pathophysiology of ASD. The discovery of significant molecular and immunological features in subgroups of individuals with ASD may allow clinicians to better stratify patients, facilitating personalized interventions and improved outcomes.

## Introduction

Autism spectrum disorder (ASD) is a heritable and prevalent neurodevelopmental disorder that is usually defined by impairments in cognition, communication and social interaction as well as by restrictive and/or stereotypical repetitive behaviors (1). Despite intense research efforts during the past decade, no definitive biological or clinical markers for ASD have been identified (2–4). This can be partly explained by the highly heterogeneous nature of ASD, both clinically and etiologically, which represents one of the greatest challenges in studying the molecular basis of ASD. The genetic underpinnings of ASD are mainly ascribed to different genetic variants such as rare copy number variations (CNVs), single-nucleotide variants (SNV) and *de novo* mutations that have been identified in ~10–20% of individuals with ASD (5–7). While hundreds of ASD-associated genes have been identified that confer varying degrees of risk, no single gene variant accounts for >1% of all ASD cases (8–10). Despite its strong genetic component, several lines of evidence suggest that environmental factors and epigenetic mechanisms may contribute to ASD etiology; however, the molecular mechanisms underlying their contributions to the development of ASD are still unclear (11, 12). Epigenetic marks, including DNA methylation (DNAm), are involved in the programming of cellular differentiation and development; it is therefore plausible that the dysregulated DNA methylation patterns caused by genetic and/or environmental factors may permanently disrupt biological pathways involved in normal brain development (13).

There is direct evidence from case-control studies showing altered targeted and genome-wide DNAm and histone acetylation in multiple tissues of affected individuals, supporting a role for epigenetic dysregulation in the development of ASD (14–18). Of note, many ASD-risk genes function as epigenetic regulators, i.e., chromatin remodelers, histone modifying enzymes and transcriptional regulators (19–22).

Many epigenetic studies have investigated ASD-associated DNAm signatures in brain tissue, in order to identify epigenetic alterations potentially causative of or mechanistically related to ASD (23–25); however, they are seriously constrained by small sample sizes and the use of autopsy-derived tissue that may be confounded by post-mortem effects on epigenetic marks. Several candidate gene-based studies revealed DNAm alterations at ASD-risk genes such as *SHANK3, OXTR, EN2*, and *MECP2* in multiple brain regions (15, 17, 18, 26). Further, genome-wide screens of DNAm in the brain of individuals with ASD have identified inconsistent differences at a variety of genomic sites; the differentially methylated CpGs were mainly associated with genes enriched in synaptic function and immune response (16, 24). Although there is sufficient evidence for immune dysregulation in individuals with ASD, immune-related genes are not among those that contain loss of function variants in next-generation sequencing studies of autistic individuals, further reinforcing the evidence for the involvement of epigenetic mechanisms in the dysregulated immune system detected in brain samples of ASD-affected individuals.

Easily accessible tissues such as blood are often used in epigenetic studies for biomarker discovery in lieu of target tissues that are difficult to access, such as brain. Recent studies have identified specific peripheral blood DNAm signatures for each of 35 neurodevelopmental/ASD syndromes caused by pathogenic variants in genes that encode epigenetic regulators (27–30). There are a very limited number of genome-wide epigenetic studies that examined DNAm changes in peripheral blood of individuals with ASD (31, 32). These studies found inconsistent evidence for DNAm alterations associated with ASD, likely due to small sample sizes (*n* < 100) and a specific focus on twin pairs with a lack of extension to the general population. A recent study by Andrews et al. (33) performed a large case-control meta-analysis of blood samples in autistic patients. This study, despite a higher sample size (*n* = 796), found no association between ASD and DNAm at genome-wide significance as no single CpG site achieved statistical significance at a Bonferroni correction level. However, they reported seven CpG sites that achieved suggestive statistical significance for association with ASD with consistent and stronger effects at the same sites among brain samples (33). Of note, these results were obtained from individuals of different ethnic backgrounds which can influence epigenetic changes as a potential confounding factor (34). Therefore, we reduced the ethnic heterogeneity by collecting the majority of samples from the same ethnicity in the present study.

In this study, we overcame previous limitations by investigating genome-wide DNAm in a large ASD (*n* = 265) cohort to identify blood-derived differentially methylated sites, as compared to control subjects. We hypothesized that, given the suggested role of epigenetics in ASD molecular etiology, epigenetic modifications could act as a useful biomarker that may contribute to the underlying etiology of subsets of patients with ASD. Our results demonstrate that DNAm alterations defined an epigenetically distinct subset of ASD cases that differentiate them from other ASD cases and controls. Notably, these observed DNAm differences were significantly associated with shifts in blood cell composition. Gene ontology analysis of the genes overlapping the differentially methylated CpG sites identified functions relevant to known pathophysiological mechanisms underlying ASD such as immune dysfunction, highlighting the biological significance of our DNAm signals.

## Methods

### Research Participants

Participants of this study were selected from existing ASD cohorts: the Province of Ontario Neurodevelopmental Disorders (POND) Network, the Simons Simplex Collection (SSC), the Autism Speaks MSSNG project and the Genome Diagnostics Laboratory at The Hospital for Sick Children (SickKids). Participants were enrolled in studies approved by the Research Ethics Boards of the respective institutions (Holland Bloorview Kids Rehabilitation Hospital, Toronto; The Hospital for Sick Children, Toronto; McMaster Children's Hospital, Hamilton; Queen's University, Kingston; Western University, London) and informed consent was obtained from participating subjects and/or their parents or guardians.

ASD study cases consisted of individuals aged 1–18 years with a primary clinical diagnosis of ASD of undefined etiology; to that end, we excluded syndromic ASD cases carrying previously identified pathogenic variants with a known effect on DNA methylation, including variants in Chromodomain Helicase DNA Binding Protein 8 (*CHD8*), Chromodomain-helicase-DNA-binding protein 7 (*CHD7*), Nuclear Receptor Binding SET Domain Protein 1 (*NSD1*), and16p11.2 deletions (21, 28, 30). Clinical diagnoses were confirmed using the Autism Diagnostic Interview-Revised (ADI-R) (35) and Autism Diagnostic Observation Schedule (ADOS) (36) or ADOS-2 (37) by clinical staff formally trained on all measures. The neurotypical control samples matched for age- and sex were selected from a collection available in our laboratory, and the SSC sample. Individuals in the control group were recruited using physician/parental screening questionnaires. The majority of individuals included in this study are of Caucasian descent (Supplementary Table 1). No significant differences were found between the case and the control group in terms of age and sex. The description of the study sample can be found in Table 1.

**Table 1:** Demographic characteristics for ASD cases and neurotypical controls.

**Sample groups**	**Control (*n* = 122)**	**Full ASD sample (*n* = 265)**	**DNAm-based ASD subset (*n* = 32)**	**Remaining ASD sample (*n* = 233)**
**Sex**	N	N	N	N
Male	84	220	27	193
Female	38	45	5	40
**Age (years)**	Mean ± SD	Mean ± SD	Mean ± SD	Mean ± SD
	12.20 ± 4	8.82 ± 4	7 ± 4.50	9.10 ± 4
**DNA collection site**	N	N	N	N
TCAG (POND/MSSNG)	6	220	26	194
SSC	30	43	6	37
Genome Diagnostics Lab (SickKids)	–	2	–	2
Weksberg Lab (SickKids)	86	–	–	–
**Cell type proportion**	Mean ± SD	Mean ± SD	Mean ± SD	Mean ± SD
B cell	0.10 ± 0.03	0.11 ± 0.04	0.16 ± 0.04	0.11 ± 0.04
CD4T	0.17 ± 0.04	0.20 ± 0.06	0.31 ± 0.06	0.18 ± 0.05
CD8T	0.10 ± 0.03	0.11 ± 0.04	0.16 ± 0.05	0.10 ± 0.04
Granulocytes	0.51 ± 0.10	0.47 ± 0.10	0.31 ± 0.05	0.50 ± 0.10
Monocytes	0.10 ± 0.02	0.08 ± 0.02	0.05 ± 0.02	0.08 ± 0.02
NK	0.05 ± 0.04	0.04 ± 0.04	0.02 ± 0.04	0.04 ± 0.04
G/L ratio	1.38 ± 0.50	1.11 ± 0.50	0.48 ± 0.10	1.20 ± 0.50
**Clinical Measures**		Mean ± SD	Mean ± SD	Mean ± SD
ADI_R: Communication domain verbal total		16.52 ± 4.60 (*N* = 200)	16.50 ± 4.30 (*N* = 22)	16.60 ± 4.60 (*N* = 178)
ADI_R algorithm total scores		43.10 ± 11 (*N* = 110)	41 ± 11 (*N* = 10)	43 ± 11 (*N* = 100)
ADOS: Communication + Social Interaction total score		13.60 ± 4.60 (*N* = 168)	14.30 ± 4.10 (*N* = 17)	13.50 ± 5 (*N* = 151)
ADOS: Social Affect total + Restricted and Repetitive Behavior total score		16.5 ± 6.10 (*N* = 52)	19 ± 5.40 (*N* = 9)	16.10 ± 6 (*N* = 43)
VABS-II: Communication Standard Score		78 ± 16.5 (*N* = 123)	80 ± 16 (*N* = 14)	76.50 ± 18 (*N* = 109)
IQ-Scale (FSIQ score)		80 ± 30 (*N* = 129)	80 ± 30 (*N* = 114)	83.60 ± 27 (*N* = 15)

*NK, Natural killer cell; G/L, Granulocyte/Lymphocyte*.

### DNAm Array Processing

DNA samples from whole blood were sodium bisulfite converted for all ASD cases and controls using the Qiagen EZ DNA Methylation kit (Qiagen, Valencia, CA) according to the manufacturer's protocol. The modified genomic DNA was then hybridized to the Illumina Infinium HumanMethylation 450 BeadChip array to interrogate over 485,000 individual CpG sites in the human genome, at The Center for Applied Genomics (TCAG), SickKids Research Institute, Toronto, Canada. The distribution of the samples on the arrays was randomized between cases and controls. Samples were run on arrays in a total of nine batches, with each batch containing ASD cases and controls.

The *minfi* Bioconductor R package (38) was used to preprocess the array data and generate Beta [β]-values from the raw intensity measures. Preprocessing included standard quality control metrics in *minfi*, including density plot, median intensity QC plots, and control probe plots. All samples passed quality control as previously described (28, 30). Methylation data were then filtered by removing probes exhibiting low detection *p*-value > 0.05 in more than 25% of the samples, cross-reactive probes, probes located on sex chromosomes, probes targeting CpG sites within 10 bp of a single-nucleotide polymorphic sites (SNPs) with a minor allele frequency > 1%, probes with raw beta = 0 or 1 in > 0.25% of samples, and non-CpG probes; a total of 427,137 probes were retained after filtering based on these criteria for normalization and downstream differential analysis. Normalization with background subtraction was then performed using Illumina control probes. The resulting β values represent percent DNAm, ranging from 0 to 1 corresponding to an unmethylated to a fully methylated CpG site.

### Differential DNAm Analysis Between ASD Cases and Neurotypical Controls

Linear regression was performed using the *limma* package (39) to identify statistically significant differentially methylated CpG sites between all ASD cases (*n* = 265) and controls (*n* = 122), accounting for covariates including age, sex, batch and estimated cell-type proportion. Blood cell type proportions were estimated using Houseman's algorithm and the Bioconductor packages *minfi* and *FlowSorted.Blood.450k* (40). Given that these cell type proportions are highly correlated, only monocyte, granulocyte, and natural killer (NK) proportions were included in the regression model (Supplementary Table 1). The remaining cell types were highly correlated with granulocyte proportion (*r* > 0.6, *p*-value < 0.05). We computed the false discovery rate (FDR) using the Benjamini-Hochberg method (41). A significant difference in DNAm between ASD cases and controls was called for each CpG site that met the cutoffs of FDR adjusted *p*-value < 0.01 and an effect size of 5% mean methylation difference (|Δβ| > 0.05).

### Functional and Genomic Enrichment of Differentially Methylated CpG Sites

For genomic enrichment analysis, the differentially methylated CpG sites were submitted to GREAT 4.0.4 (Genomic Regions Enrichment of Annotations Tool) (42) using a maximum near gene extension of 10 Kb and a hypergeometric FDR *q*-value < 0.01 for significance. Enrichment of the gene lists in each Gene Ontology (GO) term was defined against the background set of all probes that remained in the data after *minfi* probe filtering (*n* = 427,137). Terms with two or more gene hits were reported. We also compared our differentially methylated genes with known SFARI Gene ASD-risk genes (https://gene.sfari.org/) to further understand the biological relevance of our DNAm signal. In addition, we compared the genomic distribution of differentially methylated CpG sites to the background set of probes for relation to CpG island and overlapping enhancer region using a hypergeometric test (*p*-value < 0.05).

### Identification of Differentially Methylated Regions

To identify significantly differentially methylated regions (DMRs) that are associated with ASD, we used the Bioconductor bumphunter package. The bumphunting design matrix accounted for the potential confounding effects of sex, age, batch, and blood cell-type proportions (estimated monocyte, granulocytes, and NK proportions). The analysis identified consecutive CpGs no more than 0.5 kb apart with an average regional methylation difference |Δβ| > 5% between cases and controls. Statistical significance was established using 1,000 randomized bootstrap iterations. The resulting DMRs were post-filtered to retain only those with *p*-value < 0.05 across the DMR and a length (number of consecutive CpGs) of a least three sites. To further enhance stringency, we considered DMRs comprising at least one CpG from the differentially methylated CpG sites identified between ASD cases and controls, as described above.

### Comparisons of Blood Cell Composition in Sample Groups

As described above, relative proportions of underlying blood cell type were estimated using the Houseman method. We assessed our groups for possible differences in these proportions; the blood cell types measured included, B cell, CD4T, CD8T, monocyte, granulocytes, and NK. These blood cell types were those on which the Houseman method was originally trained, using peripheral blood leukocyte subtypes purchased from AllCells^®^, LLC (Emeryville, CA) or sorted cells from whole blood using negative and positive selection of surface antibodies (B-lymphocytes: CD19+; CD4 T-lymphocytes: CD3+, CD4+; CD8 T-lymphocytes: CD3+, CD8+; monocyte: CD14+; granulocytes: CD15+; natural killer: CD56+) (40). We also calculated the granulocyte/lymphocyte ratio (G/L ratio), which is a common indicator of inflammatory response. As well, the relationship between age and cell type proportion and G/L ratio was evaluated in the sample groups using the Spearman's rank correlation coefficient (*r*) and *p*-value < 0.05.

### Identification of Genetic Variants Associated With ASD

In our cohorts, we interrogated genetic variation at 366 candidate genes, selected based on the reported SFARI Gene association with ASD. These candidate genes were classified as “Category 1” (high confidence) and “Category 2” (strong candidate) by SFARI Gene at the time of manuscript submission. All the genetic variants were obtained through whole-exome and whole-genome sequencing data using the Genotypes and Phenotypes in Families (GPF) tool (https://gpf.sfari.org/) and MSSNG database (http://www.mss.ng/researchers) for individuals who underwent genome sequencing for investigation of ASD. For each gene, the mode of inheritance was specified, and rare non-synonymous variants were prioritized. For these genes, only *de novo* variants considered as likely pathogenic or pathogenic with minor allele frequency < 1% were retained as advised by the guidelines from the American College of Medical Genetics-Association for Molecular Pathology (ACMG-AMP). We then evaluated whether pathogenic *de novo* variants within ASD risk genes differed in frequency between the ASD subset and the remaining ASD cases.

To further investigate genetic factors involved in ASD, we assessed variants genome-wide (i.e., not limited to SFARI genes), including single nucleotide variants (SNVs), indels, and copy number variants (CNVs), in individuals with ASD from MSSNG (*n* = 203) and SSC (*n* = 39).

Individuals in MSSNG were sequenced using either Complete Genomics or Illumina (HiSeq 2000 or HiSeq X) platforms. SNVs, indels, and CNVs were detected from Complete Genomics samples as previously described (5), and the variants were lifted over from hg19 coordinates to hg38 coordinates for further analysis. For individuals sequenced on Illumina platforms, read alignment (hg38) and SNV/indel detection were performed using the Sentieon pipeline (43). Individuals in SSC were sequenced on the Illumina HiSeq X platform. SNVs and indels were downloaded from SFARI Base (https://www.sfari.org/resource/sfari-base). SNVs and indels from both cohorts were annotated using a custom ANNOVAR-based pipeline (44). CNVs were detected in both MSSNG (Illumina samples only) and SSC using a previously-described workflow (45) involving the algorithms ERDS (46) and CNVnator (47). High-confidence *de novo* SNVs and indels were detected using DeNovoGear (48) as previously described (5). *De novo* CNVs were identified as those that were detected by both ERDS and CNVnator, that were rare [ <1% frequency in MSSNG parents and 1,000 Genomes Project (49) population controls], and that were not detected by ERDS or CNVnator in either parent.

We compared our ASD groups for possible differences in the number of *de novo* SNVs/indels per individual, and frequency or type of variants (for SNVs and indels: stop gain/ splice site/ frameshift; for CNVs: deletion/duplication).

### Examination of Risk Factors and Clinical Phenotypes

See Table 2 for descriptions of all clinical measures.

**Table 2:** Clinical measures analyzed in our ASD cohort.

**Scale**	**Subscale**	**The age range analyzed**
WASI, WASI II, WISC IV or SB-5	Full Scale Intelligence Quotient	6–18 years 1–6 years
ADI-R	Communication domain verbal total	2–18 years
	Algorithm total scores [in three domains: social interaction, communication, and restricted repetitive behavior (RRB)]	2–18 years
ADOS	Communication + Social Interaction total score	2–18 years
	Social Affect + Restricted Repetitive Behaviors total score	2–18 years
VABS-II	Communication Standard Score	1–6 years

The age range represents the group of ages administered to each clinical measure.

*WAS, Wechsler Abbreviated Scale of Intelligence; WISC, Wechsler Intelligence Scale for Children; SB, Stanford Binet Intelligence Scales*.

We assessed risk factors previously shown to be associated with an increased risk of ASD. These included assisted reproductive technology (ART), gestational age, maternal smoking, parental age at the time of birth, and proband sex. Clinical phenotypes were measured by the following scales: Vineland Adaptive Behavior Scales (VABS-II); ADIR and ADOS as indicators of ASD severity and symptomatology. IQ was assessed using the appropriate scale as determined by the child's age: the Wechsler Abbreviated Scale of Intelligence (WASI, WASI-II), the Wechsler Intelligence Scale of Children-IV (WISC-IV), or the Stanford Binet Intelligence Scales, 5th ed (SB-5) (FSIQ score used for each test). These measures, risk factors and clinical data were assessed against DNAm in all samples for which they were available, namely ASD cases recruited from the POND network, MSSNG and SSC. Of note, these clinical features were measured in the same study visit as tissue sample collection, or within a 7-month period.

## Results

### Identification of DNAm Alterations Associated With ASD

**Figure 1:**
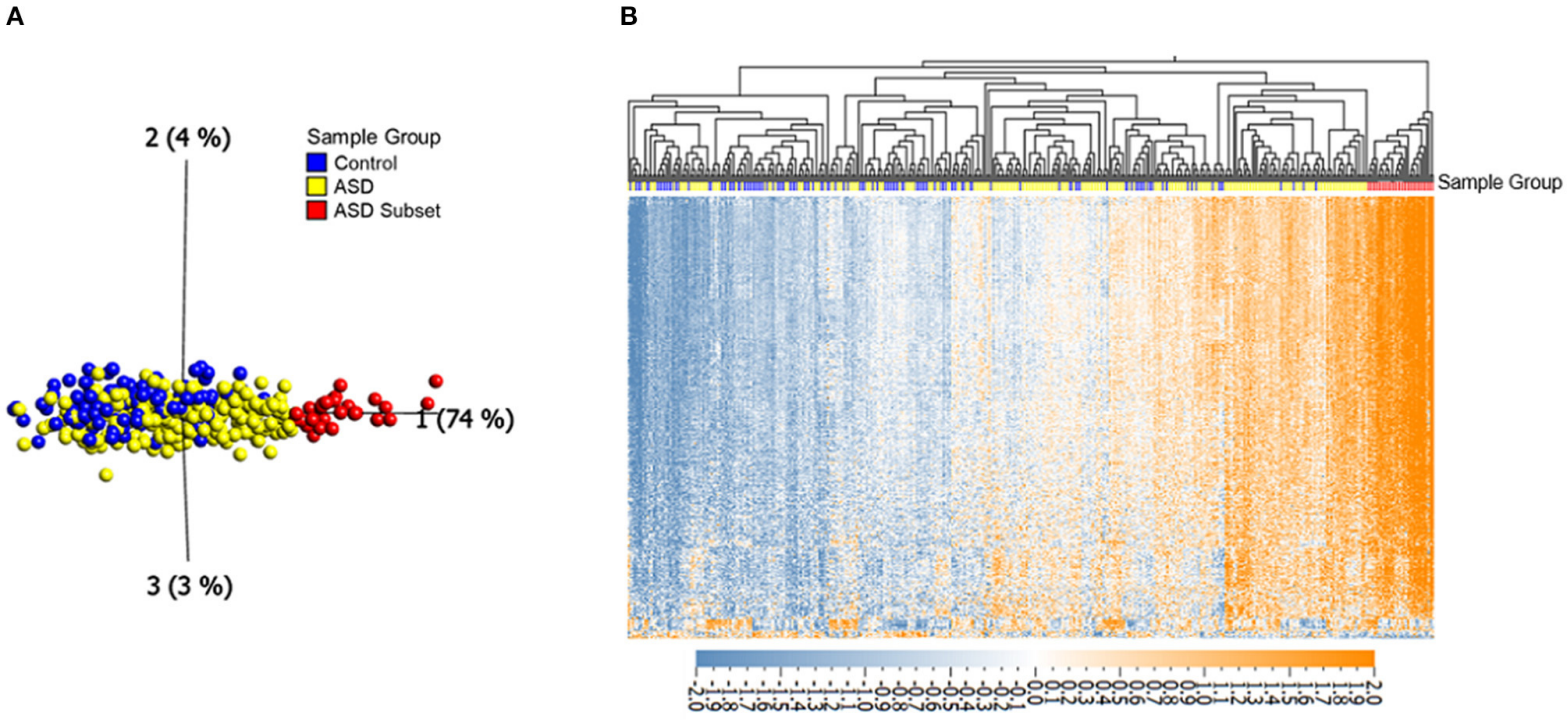
Differential DNAm at 400 in ASD (*n* = 265) and neurotypical controls (*n* = 122) reveals an epigenetically unique subset of ASD cases (*n* = 32). **(A)** Principal component analysis performed on 400 CpGs (FDR adjusted *p*-value < 0.01 and |Δβ| > 5%), with axes representing first three principal components. **(B)** Corresponding heatmap hierarchical clustering using Eucledian distance metrics. Orange indicates high DNAm, and blue gray indicates low DNAm, normalized for visualization (mean = 0, variance = 1). Samples labeled with red and yellow represent the ASD subset and the remaining ASD cases, respectively, blue samples represent controls.

To investigate DNAm alterations associated with ASD, we compared genome-wide DNAm in blood from individuals with heterogeneous ASD (*n* = 265) to sex- and age-matched, neurotypical controls (*n* = 122) at 427,137 CpG sites (Table 1). We identified 400 significantly differentially methylated CpG sites across the genome at an FDR adjusted *p*-value < 0.01 and |Δβ| > 5% (5% methylation difference) as illustrated by a volcano plot in Supplementary Figure 1 and Supplementary Table 2. Ninety seven percentage of these CpG sites exhibited higher methylation levels (with DNAm differences ranging from 5 to 14%) in ASD cases as compared to controls. Principal component analysis (PCA) run on the 400 differentially methylated sites showed a gradient of DNAm values starting from a group of mostly controls to a separate subset of ASD-affected individuals that was epigenetically distinct from both controls and remaining ASD cases (*n* =32; Figure 1; Table 1). This observation was supported by hierarchical clustering of DNAm values, with this DNAm gradient going from a cluster comprised of mostly controls where the majority of differentially methylated CpGs are hypomethylated to the distinct ASD subset where the majority of these sites are hypermethylated. No significant differences were found between the ASD subset and the remaining ASD cases in terms of age and sex (Mann-Whitney *p*-values > 0.05); both groups of ASD, the subset and remaining cases, included individuals from all three cohorts (POND, MSSNG and SSC; see Table 1 and “Research participants” subsection of **Methods** for details). Moreover, running an additional *limma* regression model with DNA collection site included as an additional covariate did not alter the results; of the 400 significantly differentially methylated sites, 381 CpGs remained significant and could still differentiate the ASD subset from the remaining ASD cases (results not shown).

**Figure 2:**
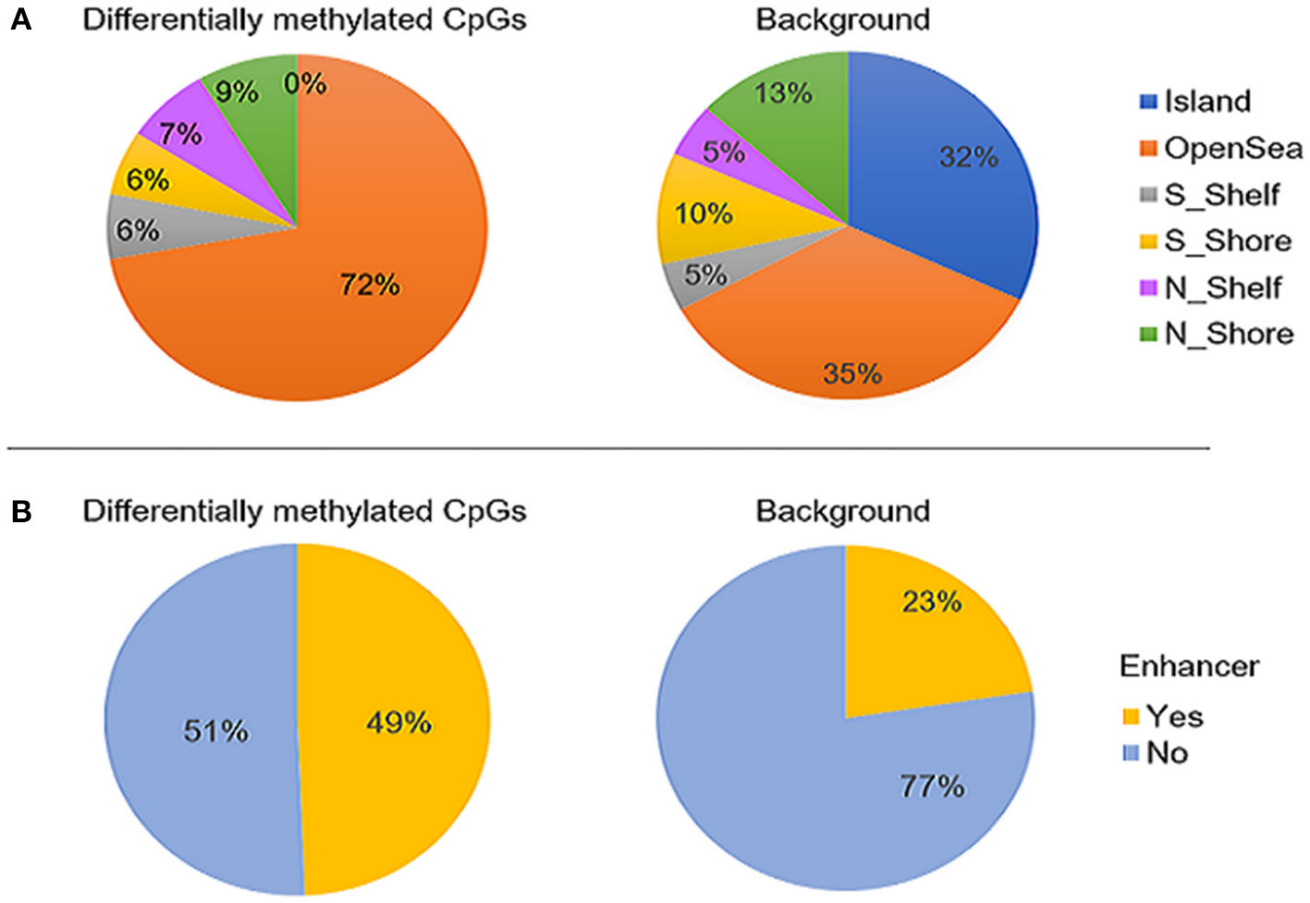
The genomic distribution of the 400 differentially methylated CpG sites identified between ASD cases (*n* = 265) and controls (*n* = 122, left) compared to the background set of all probes that retained after probe filtering (*n* = 427,137) (right). **(A)** proportion CpG sites in relation to CpG islands and **(B)** proportion of CpGs overlapping enhancer regions. The differentially methylated sites were found to be significantly enriched in open sea and enhancers (*p*-values < 0.05) and depleted in CpG islands (*p*-value < 0.01). “Island” is CpG island; N_shore and S_shore are north (upstream) and south (downstream) shores, i.e. 2kb regions flanking island; N_shelf and S_shelf are north (upstream) and south (downstream) shelves, i.e. 2kb regions flanking island shores.

The majority of the differentially methylated CpG sites mapped to promoter regions or gene bodies of 159 RefSeq genes (Supplementary Table 2). The genomic distribution of the 400 CpGs was compared with that of the background test sites. We found a significantly higher proportion of CpGs located in open sea (72 vs. 35%; *p*-value < 0.05) and a depletion of island CpGs (0 vs. 32%; *p* < 0.01) (Figure 2A). Moreover, the differentially methylated sites were found to be enriched in enhancers, as compared to the full 427,137 CpG sites (49 vs. 23%; *p*-value < 0.05) (Figure 2B).

### Functional Enrichment of Differentially Methylated CpG Sites

We performed gene ontology analysis of the 400 differentially methylated sites using GREAT 4.0.4 to assess enrichment of common biological processes, molecular functions, and cellular components of genes mapping to these CpG sites. GREAT identified 159 genes that overlapped the 400 CpG sites detected in our differential methylation analysis. We identified 27 GO biological processes assigned to the differentially methylated sites; the majority of them were related to immune function, such as immune and inflammatory response, in addition to enrichment for cellular secretion, as the top GO terms (hypergeometric FDR *q*-value < 0.01; Supplementary Table 3). Two GO cellular components met significance: granule membrane and inflammasome complex (Supplementary Table 4), while no molecular functions were significantly enriched.

Of the genes mapping to sites of differential methylation (*n* = 159), 26 are listed by SFARI as ASD-risk genes (Supplementary Table 5). These included genes encoding proteins of the immune and nervous systems, such as Cullin 3 (*CUL3* [MIM: 614496]), ANNEXIN A1 (*ANXA1* [MIM:151690]), SH3 and multiple ankyrin repeat domains 2 (*SHANK2* [MIM: 613436]) and MET protooncogene (*MET* [MIM: 164860]).

### Identification of Differentially Methylated Regions

In addition to assessing each CpG independently, DMRs were evaluated, identifying regional DNAm differences. Significant DMRs were defined by *p* < 0.05, |Δβ| ≥ 5% and a length of at least three consecutive CpG sites, of which at least one had already been identified as a significant site in our differential methylation analysis (400 sites). Regional DNAm analysis identified 15 significant DMRs (Supplementary Table 6). As expected, these CpGs did not include those that mapped to open sea but rather higher density CpG regions including island shelves and shores. The longest DMR spanned 0.67 Kb and mapped to Galactoside-Binding, Soluble, 1 (*LGALS1*) which encodes Galectin-1 involved in regulating apoptosis, cell proliferation and cell differentiation (Supplementary Table 6).

### Assessment of Blood Cell Type Composition in DNAm-Based Sample Groups

**Figure 3:**
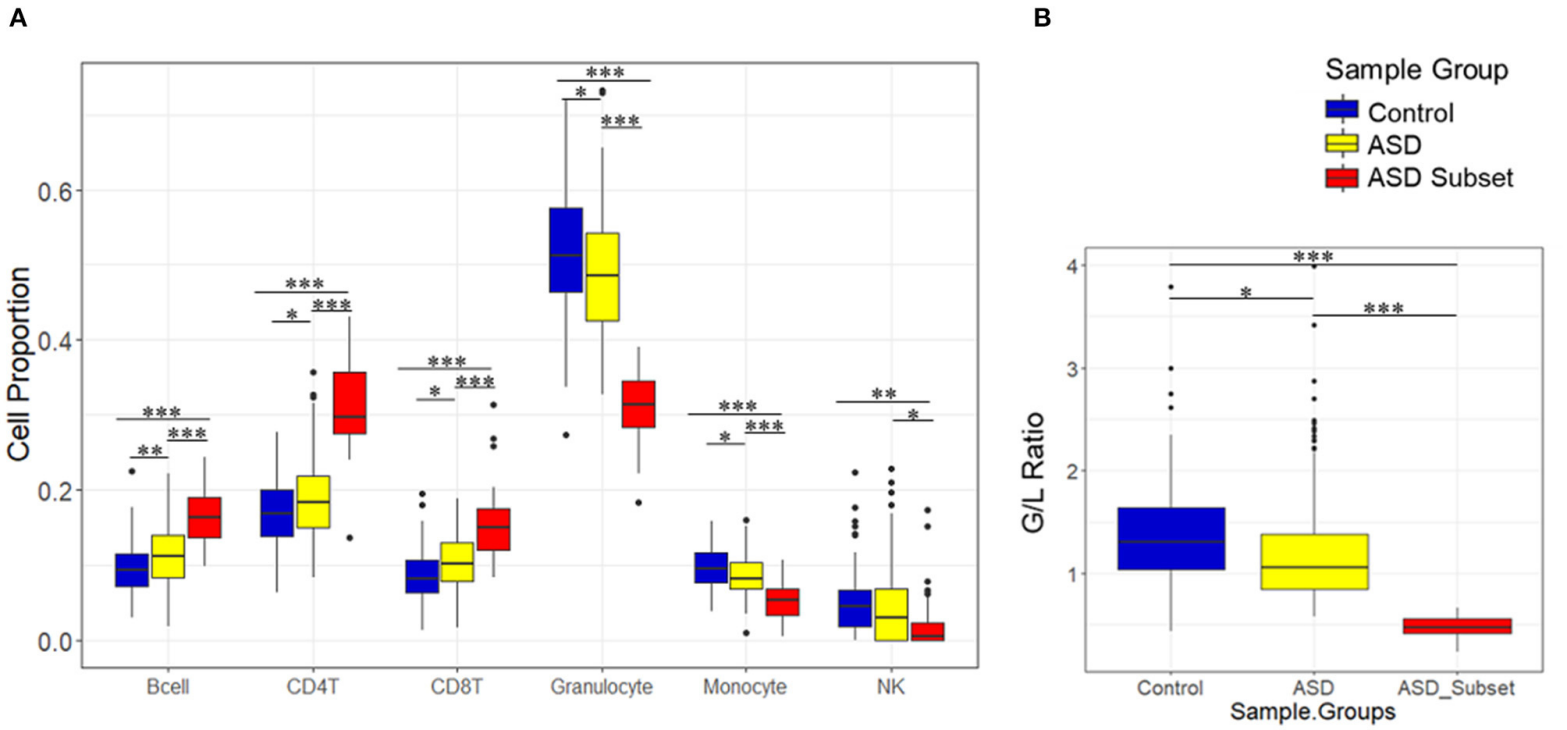
Relative proportions of blood cell types in sample groups, as estimated by DNAm. Boxplots show immune blood cell proportions estimated by the Houseman method **(A)** and calculated granulocyte/lymphocyte (G/L) ratio **(B)**. Epigenetically unique ASD subset (red; *n* = 32), the remaining ASD cases (yellow; *n* = 233), and controls (blue; *n* = 122). ASD subset exhibited significant shifts in cell type proportions and the G/L ratio (*p*-value < 0.01) as compared to the remaining ASD cases and controls. Black bars with asterisk represent significant differences in estimated blood cell proportions between the groups (*p ≤ 0.05; **p ≤ 0.01; ***p ≤ 0.001).

**Figure 4:**
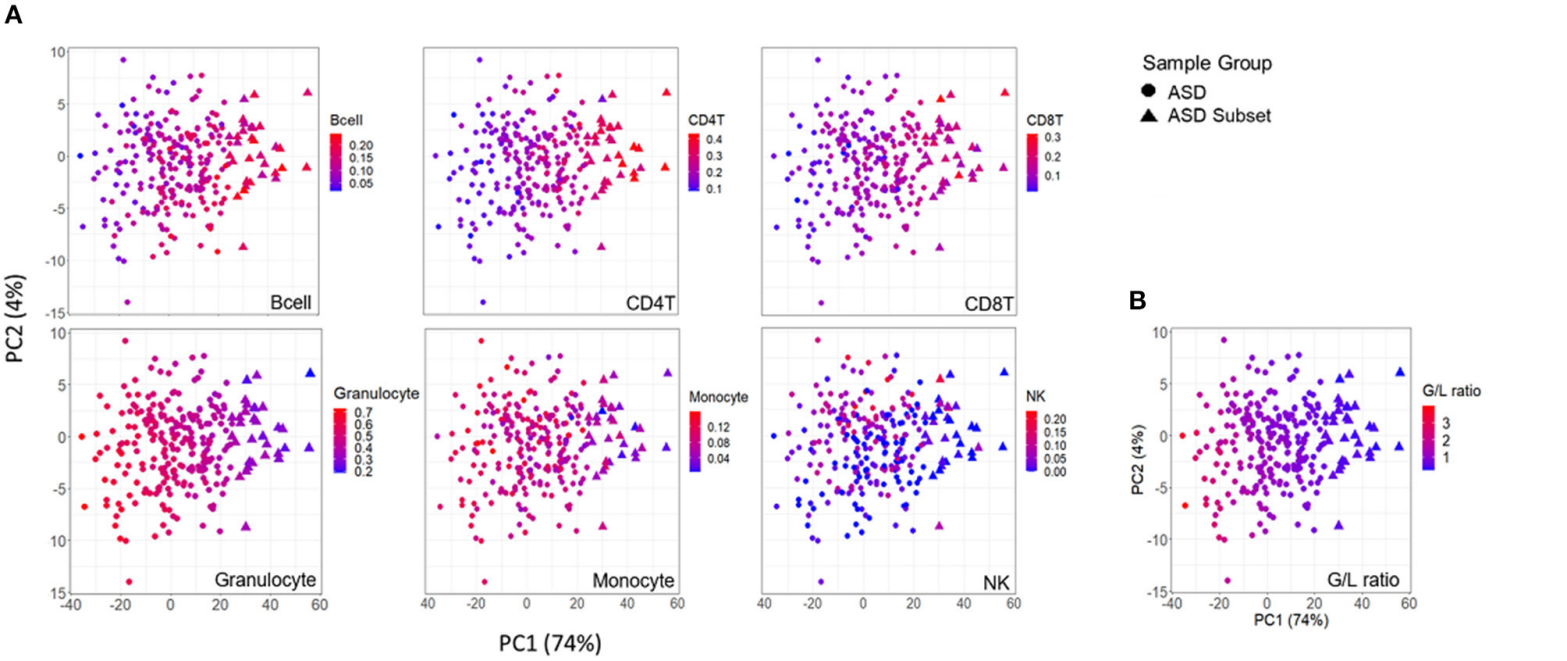
Association between DNAm variation and cell type proportions across ASD cases. Scatterplots of first two principal components from principal component analysis (PCA) performed on 400 differentially methylated sites between ASD cases (*n* = 265), and controls (*n* = 122). **(A)** distribution of blood cell proportions [from left to right: B cells, CD4T cells, CD8T cells, granulocytes, monocytes and natural killer cells (NK)] and **(B)** granulocyte/lymphocyte (G/L) ratio across ASD cases. Samples plotted as triangles represent distinct ASD subset (*n* = 32) and circles represent remaining ASD cases (*n* = 233). Color of point indicates proportion of given cell type in each sample.

To investigate the relationship between the identified enrichment in immune function and inflammatory response associated with the ASD subset signature, we evaluated possible differences in estimated immune blood cell proportions between the three groups: the epigenetically unique ASD subset, the remaining ASD cases, and controls. Interestingly, we identified shifts in cell type proportions in the ASD subset as compared to the remaining ASD cases and controls (Figures 3, 4). Namely, these individuals exhibited a significant increase in CD4T proportion (*p*-value < 0.01). In contrast, granulocyte proportion significantly decreased and accordingly, the G/L ratio was lower (*p*-value < 0.0001) in the ASD subset. We found significant differences in blood cell type composition between the remaining ASD cases and controls (Table 3). In addition, we ran 20 iterations of Mann-Whitney tests for each cell type on randomly sampled groups of *n* = 32 ASD cases (from the ASD cases excluding the DNAm-based subgroup; *n* = 233) vs. the remaining ASD cases, to ensure the true differences held up to permutation testing. Only a single iteration produced a *p*-value < 0.05 (seen in monocytes) and no permutation analysis ed *p*-values approached neared those of the true associations (all *p*-values > 0.04; Supplementary Figure 2).

**Table 3:** Immune blood cell composition comparisons between sample groups: the epigenetically unique ASD subset (*n* = 32), the remaining ASD cases (*n* = 233), and controls (*n* = 122).

**Cell type composition**	**Comparison groups**		
	**ASD subset vs. ASD**	**ASD subset vs. Control**	**ASD vs. Control**
	**mean difference ± SE**	**mean difference ± SE**	**mean difference ± SE**
B cell	0.05 ± 0.01^***^	0.07 ± 0.01^***^	0.02 ± 0.003^**^
CD4T	0.13 ± 0.01^***^	0.14 ± 0.01^***^	0.01 ± 0.005^*^
CD8T	0.06 ± 0.01^***^	0.07 ± 0.01^***^	0.01 ± 0.003^*^
Granulocytes	−0.20 ± 0.01^***^	−0.21 ± 0.01^***^	−0.01 ± 0.01^*^
Monocytes	−0.03 ± 0.004^***^	−0.04 ± 0.003^***^	−0.01 ± 0.002^*^
NK	−0.02 ± 0.01^*^	−0.03 ± 0.01^**^	−0.01 ± 0.004
G/L ratio	−0.72 ± 0.03^***^	−0.92 ± 0.05^***^	−0.18 ± 0.05^*^

NK, Natural killer cell; G/L, Granulocyte/Lymphocyte.

* p ≤ 0.05;

** p ≤ 0.01;

*** *p ≤ 0.001*.

**Figure 5:**
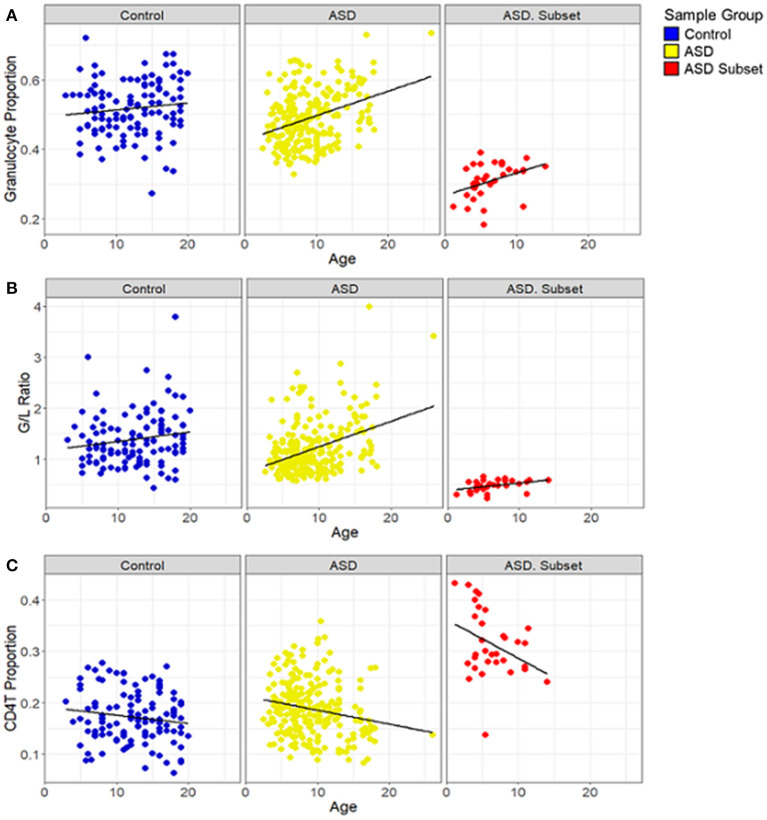
Relationship between blood cell proportions and sample age in individuals with ASD. Box plots depict **(A)** granulocyte proportion, **(B)** granulocyte/lymphocyte (G/L) ratio and **(C)** CD4T proportion in samples plotted against age. ASD subset (red; *n* = 32), the remaining ASD cases (yellow; *n* = 233), and controls (blue; *n* = 122). In all both ASD groups, age was positively correlated with the granulocyte proportion (ASD subset: *r* = 0.43, *p*-value = 0.01; the remaining ASD: *r* = 0.35, *p*-value < 0.001) and the G/L ratio (ASD subset: *r* = 0.45, *p*-value = 0.01; the remaining ASD: *r* = 0.37, *p*-value < 0.001) and negatively correlated with CD4T (ASD subset: *r* = −0.4, *p*-value = 0.02; the remaining ASD: *r* = −0.2, *p*-value = 0.002); the remaining ASD: *r* = −0.2, *p*-value = 0.002). In controls, no significant correlation was found between age and the blood cell compositions.

To investigate if the cell-type proportions was correlated with the age of the samples, we performed correlation analysis based on Pearson correlation coefficient (*r*) and a *p*-value < 0.05. We found that in all the ASD cases, age was positively correlated with granulocyte proportion (ASD subset: *r* = 0.43, *p*-value = 0.01; the remaining ASD: *r* = 0.35, *p*-value < 0.001) (Figure 5A) and G/L ratio (ASD subset: *r* = 0.45, *p*-value = 0.01; the remaining ASD: *r* = 0.37, *p*-value < 0.001) (Figure 5B). A significant negative correlation was found between age and CD4T proportion in the ASD cases (ASD subset: *r* = −0.4, *p*-value = 0.02; the remaining ASD: *r* = −0.2, *p*-value = 0.002) (Figure 5C). In contrast, the cell type proportions, and the G/L ratio showed no correlation with age in control subjects (*r* < 0.2, *p*-value > 0.5).

### Reassessment of Differential Methylation Associated With ASD After Removing the ASD Subset

To further investigate DNAm in our ASD cohort, we removed from our dataset the 32 ASD cases that were detected as unique both epigenetically and in blood cell composition and performed differential methylation analysis between the remaining ASD cases (*n* = 233) and controls (*n* = 122) using the same analytical methods, i.e., *limma* regression, covarying for age, sex, and estimated cell type proportion (granulocyte, NK, and monocyte). Linear regression analyses identified 77 significantly differentially methylated CpG sites with FDR adjusted *p-*value < 0.01 and |Δβ| > 5% (Supplementary Table 7). Notably, PCA continued to show a gradient of DNAm value tightly associated with granulocytes proportion and the G/L ratio across the ASD cases on PC1 (Supplementary Figures 3, 4). As such, epigenetic differences between ASD cases and controls may be attributed either to underlying differences in blood cell composition or the identification of CpG sites that are blood cell type specific.

### Identification of Genetic Variants Associated With ASD

We investigated 366 genes classified by SFARI Gene as high risk for ASD and looked for differences in pathogenic *de novo* variant frequency between our DNAm-based ASD groups. In the ASD subset, we identified six individuals (18%) with nine different *de novo* pathogenic variants at different genes (Supplementary Table 8). Only 34 individuals (14%) of the remaining ASD cases harbored *de novo* pathogenic variants (Supplementary Table 9); most of these were missense variants. Given that the ASD subset did not differ from the rest of the ASD cases in the frequency or type of high-risk variants, we, therefore, expanded our analysis to identify the genome-wide *de novo* pathogenic variants including SNVs, indels, and CNVs associated with ASD.

Likewise, no significant differences were detected between these two groups of ASD for the average number of *de novo* SNVs/indels per individual (average of 1.2 per individual in both groups), and frequency or type of SNVs and indels. However, in the larger ASD cohort, i.e., not the subset with unique DNAm, we identified nine overlapping CNV regions of mostly duplications that were identified in more than one patient; The only common deletion identified was assigned to the gene Patched domain-containing protein 1 (*PTCHD1* [MIM: 300828]), which is a high -risk ASD gene (Supplementary Table 10).

### Examination of Risk Factors and Clinical Phenotypes in DNAm-Based Groups

We evaluated the factors that increase the risk of ASD and behavioral phenotypes, comparing the epigenetically unique ASD subset to the remaining ASD cases. No significant differences were detected between these two groups of ASD cases for ART, gestational age, maternal smoking, parental age, and proband sex (*t*-test *p*-values > 0.05). Similarly, clinical phenotypes measured in the cohort did not differ significantly between individuals of the two groups of ASD. Namely, no significant differences were detected between these two groups of ASD cases for ADI-R (Communication domain verbal and algorithm total), ADOS (Communication + social interaction and social Affect + restricted repetitive behaviors) and VABS-II (Communication Standard), and IQ scores (FSIQ) (Table 1).

## Discussion

In this study, we sought to assess genome-wide DNAm alterations associated with ASD. We identified a subset of ASD cases that exhibit differential methylation patterns distinct from both controls and the remaining ASD group as well as significant shifts in cell type proportions, i.e., the granulocyte-to-lymphocyte ratio was significantly lower in the ASD subset than in the remaining ASD cases and controls. In the present study, beyond blood cell composition, we found no significant differences between the ASD subset and the remaining ASD cohort, including sex, age, genetic risk variants or clinical measures such as ADI, VABS and ADOS subscale scores. Furthermore, our study provides additional support for previously reported involvement of *SHANK2, ANXA1, MET, CUL3* and other genes in the pathophysiology of ASD. Our study suggests that at least one mechanism underpinning differential methylation between ASD cases and neurotypical controls is a difference in blood cell type proportion.

It is important to note that blood cell type proportion was estimated from DNA methylation. As such, it is possible that differences observed may be attributed to true changes in the blood cell composition or that DNA methylation alterations exist in the ASD subgroup at CpG sites used to estimate blood cell composition. A previous meta-analysis of methylation studies of ASD by Andrews et al. (33) reported in their patient demographics, significant differences in granulocyte and B cell proportions between ASD cases and control subjects parallel to those found in our study. These investigators found no single CpG to meet genome-wide significance using Bonferroni correction (*p* < 1.12 × 10^−7^) for the association between ASD and DNAm and did not interpret cell types differences in the discussion (33). Regardless, it is plausible that these methylation alterations may be indicative of altered immune function in the ASD subset. As well, this is not the first instance in which DNAm has been used to identify changes in blood cell proportion associated with a disorder; this has been reported in both asthma and systemic lupus erythematosus by Kong et al. (50). They showed that the proportion of DNAm alterations attributable to changes in cell type composition varies considerably in both asthma and systemic lupus erythematosus, suggesting disease-specific cell subtype proportion changes contributing to DNAm alterations (50). Future studies using differential blood counts and DNAm-based blood cell estimates from the same blood draws are required to clarify this relationship.

Two additional findings in our study support the observed relationship between DNAm, immune cell type and ASD in our cohorts. The majority of the differentially methylated CpG sites were enriched for gene ontology categories implicated in immune and inflammatory response (Supplementary Table 4), which strengthens the scientific evidence that a dysregulated immune system is one of the contributing factors in ASD (51). Our findings are consistent with the methylation analysis in brain that demonstrates altered immune response in the cortical region of autistic cases, Brodmann area 10, correspond with epigenetic modulation of genomic regions relevant to several categories related to immune response, including inflammatory response to antigens and positive regulation of cytokine biosynthetic processes (16). As well, brain and blood transcriptome studies show that many of the genes exhibiting a higher variability in their overall expression pattern were related to the immune system in autistic individuals, indicating dysregulation in immune functions in ASD (52–55). A comparison between our differentially methylated sites and the transcriptomic data reported in gene expression studies showed overlap with two genes characterized by significant DNA hypermethylation in our ASD subset and decreased expression levels. The *ANXA1* gene, which encodes a protein that functions in adaptive immunity, was found to be upregulated in ASD individuals (56). As well, ankyrin repeat domain 22 (*ANKRD22*) overlapping three differentially methylated CpGs was found to be significantly downregulated in blood (57); this gene encodes a protein that specifically interacts with STING, a critical protein function in multiple anti-viral innate immune pathways (58). The prior evidence of hypermethylation of the immune-related genes correlating with decreased expression further supports the potential role of epigenetic regulation of the altered immune response associated with ASD. Further, when the genome-wide linear regression of ASD vs. controls was rerun with the ASD subset removed, the differentially methylated CpG sites were still strongly predictive of blood cell proportion.

We searched for overlap between differentially methylated sites in our blood DNAm and previous studies of DNAm in individuals with ASD. We found 23 overlapping CpG sites with DNAm signature detected in adult cortical regions (16); CpG sites overlapped notable genes such as syndecan-2 (*SDC2* [MIM:142460]), Dystonin (*DST* [MIM: 113810) and mediator complex subunit 12L (*MED12L* [MIM: 611318]); However, we did not find any overlap with the findings of blood DNAm studies (31, 32).

In recent years, there is emerging evidence and growing concern that a dysregulated or abnormal immune response may be involved in the development of some forms of ASD. Several lines of research have provided substantial evidence of immune dysregulation in subsets of individuals with ASD, including skewed inflammation responses, cytokines, and total numbers and frequencies of immune cells (59–61). The inconsistencies in previous research findings are marked by considerable variation in the prevalence of ASD by ethnicity/race, sex, geographic area, and level of intellectual ability. The heterogeneity of ASD is the source of much difficulty in study underlying pathophysiology, etiology and biomarkers of this neurodevelopmental disorder. This is especially apparent in genetic studies of ASD, in which rare SNVs and CNVs account for only a small proportion of ASD risk. Biological measures, such as DNAm or immune markers, which exhibit consistent changes across substantial subsets of individuals with ASD, may provide a new avenue for ASD research.

A number of limitations should be noted. As described above, cell types composition was not measured directly but rather estimated using specific CpG sites that exhibit blood cell-type specific DNAm patterns. While this method has been validated and is widely used, it is possible that DNAm levels at these CpGs sites were altered by factors other than cell type, causing skewed estimates. However, it is worth noting that cell type of origin is one of the strongest predictors of DNAm patterns. For example, DNAm patterns from a single tissue sampled from two individuals are more strongly correlated than patterns from a single individual in different tissues. Beyond this, risk factors and clinical phenotypes were available for only a subset of ASD individuals in this study, which may considerably reduce the statistical power to detect associations between DNAm and behavioral phenotype. This missing information is also important as it may have contributed to an unbalanced study design. The distribution of our nominal *p*-values suggested genomic inflation (see QQ-plot in Supplementary Figure 5), which is associated with inflated false positives. Although this can be result of an unbalanced study design or confounding factors that are not accounted for in the statistical model, it may also result from a strong association between DNAm and ASD status at a large number of CpGs sites (62). We propose that by testing the effect of systematic blood cell composition differences, as observed between our cohorts, on DNAm changes we expect broad, genome-wide differences with large effect sizes that would mimic genomic inflation. Furthermore, we accounted for important covariates, including technical factors, blood cell proportions, sex, age, etc. and only reported CpGs that met stringent significance threshold of FDR adjusted *p*-value < 0.01 and Δβ| > 0.05. Nonetheless, this observation which can sometimes reflect genomic inflation does support the need for independent replication of these findings in future to better understand the relationship between ASD, blood cell composition and DNAm levels.

## Conclusion

In summary, this study demonstrates a gradient of DNAm alterations across our ASD cases tightly associated with shifts in immune cell type proportions. Moreover, we report an epigenetically unique subset of ASD cases that exhibited a significant difference in immune cell type proportions, as compared to the controls and the remaining ASD cases. Our findings build on past reports of changes in the immune systems of children with ASD, supporting the potential role of altered immunological mechanisms in the complex pathophysiology of ASD. The discovery of significant molecular and immunological features in subgroups of individuals with ASD provides unique insight into the molecular pathophysiology of ASD that can help clinicians to better stratify patients, facilitating personalized interventions and improved outcomes. These results may lead to the hypothesis that immunological shifts may induce long-term changes through modulation of DNA methylation in genomic regions involved in the immune response, such as the hypermethylated regions observed in the subset of ASD cases in our data.

## Data Availability Statement

The datasets generated for this article are not readily available because the consents obtained did not cover open access/sharing of the data. Requests to access the datasets should be directed to Rosanna Weksberg, rweksb@sickkids.ca.

## Ethics Statement

The studies involving human participants were reviewed and approved by Research Ethics Board (REB), The Hospital for Sick Children, Toronto, Canada. REB number: 0019980189. Written informed consent to participate in this study was provided by the participants' legal guardian/next of kin.

## Author Contributions

MJ and SGo analyzed and interpreted the data and wrote the manuscript. SC participated in study design and interpreted the data. BT generated genomic variant data. SS collected patient samples and performed genome-wide sequencing to identify variants. EK, MA, RN, SGe, JC, RS, SS, and EA are members of the executive committee of the POND Network, which provided patient cohorts and phenotype data. EG assisted with the interpretation of immune cell type data and provided input on study design. RW is the principal investigator and was involved in all aspects of the study. All co-authors edited and revised the manuscript.

## Conflict of Interest

The authors declare that the research was conducted in the absence of any commercial or financial relationships that could be construed as a potential conflict of interest.

## Abbreviations

ADI-R: autism diagnostic interview revised
ADOS: autism diagnostic observation schedule
ANXA1: ANNEXIN A1
ANKRD22: ankyrin repeat domain 22
ASD: Autism spectrum disorder
SB-5: Stanford Binet Intelligence Scales
CHD7: Chromodomain-helicase-DNA-binding protein 7
CHD8: Chromodomain Helicase DNA Binding Protein 8
CNV: Copy number variant
CpG: Cytosine- phosphate- Guanine
CUL3: Cullin 3
DMR: Differentially methylated region
DNAm: DNA methylation
FDR: False Discovery Rate
G/L Ratio: granulocyte/lymphocyte ratio
GO: Gene ontology
MET: MET protooncogene
NK: Natural killer cell
NSD1: Nuclear Receptor Binding SET Domain Protein 1
PCA: Principal component analysis
REB: Research ethics board
SFARI: Simons Foundation Autism Research Initiative
SHANK2, H3: multiple ankyrin repeat domains 2
SNV: single nucleotide variant
VABS-II: Vineland Adaptive Behavior Scales
WAS: Wechsler Abbreviated Scale of Intelligence
WISC: Wechsler Intelligence Scale of Children.
