# Supplementary material for: An Epigenetically Distinct Subset of Children With Autism Spectrum Disorder Resulting From Differences in Blood Cell Composition
Source: Front Neurol. 2021 Apr 16;12:612817. doi: 10.3389/fneur.2021.612817 (PMC8085304; doi:10.3389/fneur.2021.612817)
Supplement: Supplementary file 2 [file Image_1.PDF]

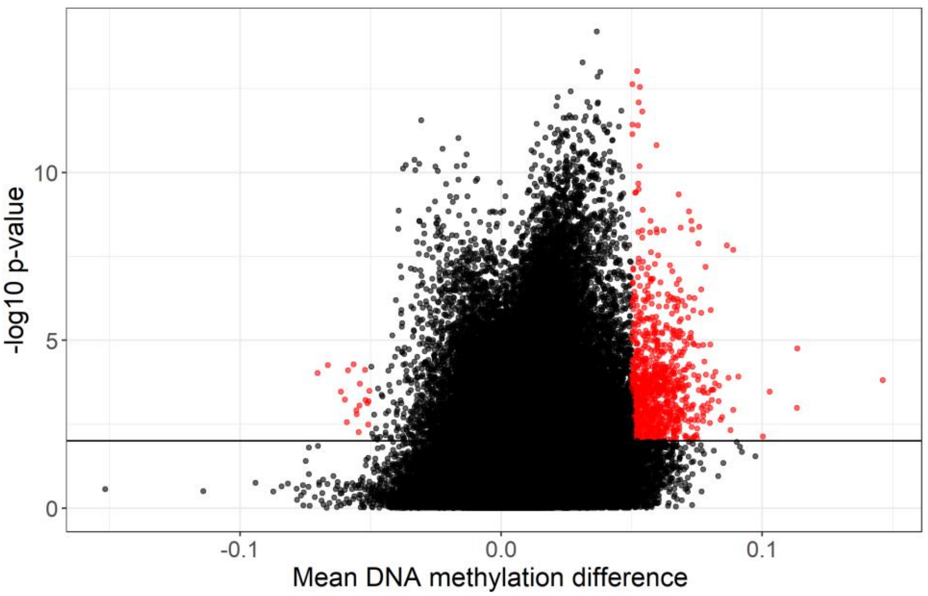

Figure S1: Volcano plot displays mean DNA methylation difference between ASD and controls at genome-wide CpG sites vs. −log10 (unadjusted *p*-value) after adjustment for batch, age, sex, and cell type proportion. Significant differentially methylated CpG sites (n = 400) with FDR q-value < 0.01 and |Δβ| > 0.05 are presented in red. The black horizontal line indicates cut-off for significance threshold (FDR adjusted *p*-value < 0.01).
