# Supplementary material for: An Epigenetically Distinct Subset of Children With Autism Spectrum Disorder Resulting From Differences in Blood Cell Composition
Source: Front Neurol. 2021 Apr 16;12:612817. doi: 10.3389/fneur.2021.612817 (PMC8085304; doi:10.3389/fneur.2021.612817)
Supplement: Supplementary file 3 [file Image_2.PDF]

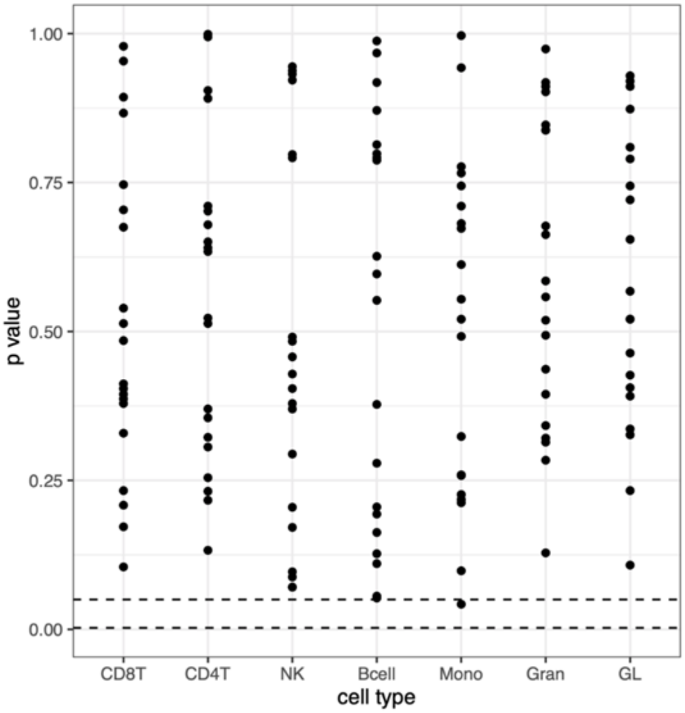

Figure S2. Plot of *p*-values generated from 20 iterations of randomly sampling groups of n=32 ASD cases (from the ASD cases excluding the DNAm-based subgroup; n=233) vs. the remaining ASD cases for each cell type. The Top and lower dashed lines indicate cut-off for significance threshold of *p*-value<0.05 and p<0.0025 (FWER), respectively. Only a single iteration produced a p-value<0.05 (seen in monocytes) and no permuted *p*-values neared those of the true associations (all p-values>0.04).
