# Supplementary material for: An Epigenetically Distinct Subset of Children With Autism Spectrum Disorder Resulting From Differences in Blood Cell Composition
Source: Front Neurol. 2021 Apr 16;12:612817. doi: 10.3389/fneur.2021.612817 (PMC8085304; doi:10.3389/fneur.2021.612817)
Supplement: Supplementary file 4 [file Image_3.PDF]

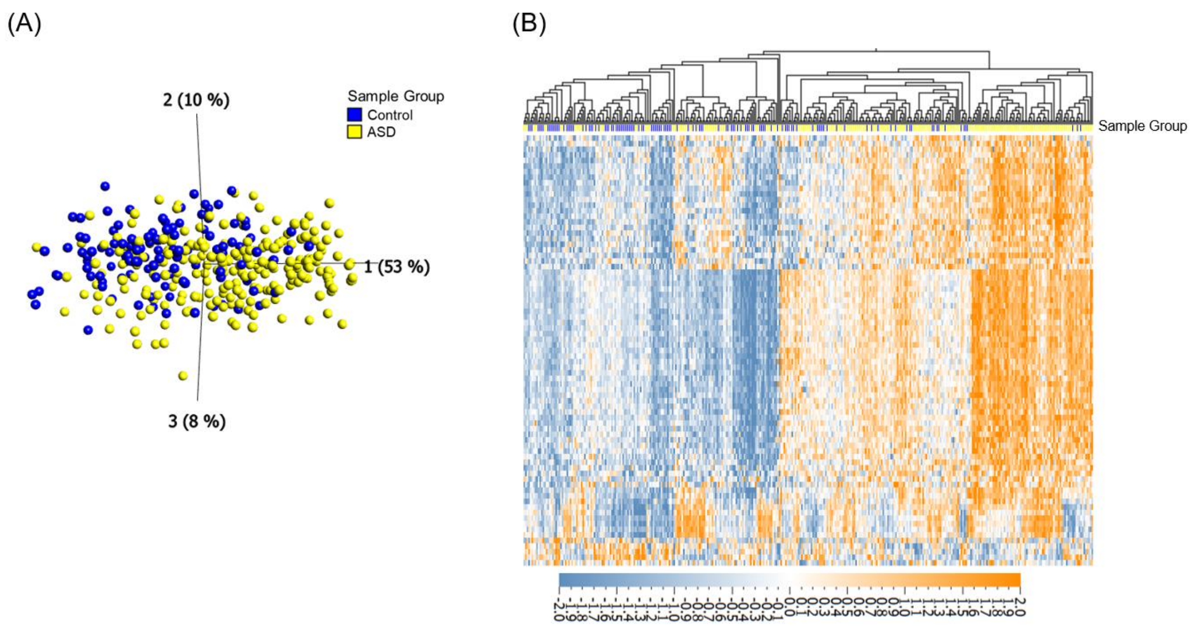

Figure S3. Reassessment of differential methylation associated with ASD after removing the 32 unique ASD cases, identified 77 differentially methylated CpG sites (FDR adjusted *p*-value<0.01; $|\Delta\beta| > 5\%$.). (**A**) Principal component analysis (PCA, first three principal components labelled) plot and (**B**) corresponding hierarchical clustering by Eucledian distance metrics, Samples labelled with yellow represent the ASD cases and blue samples are control subjects. On the heatmap, orange indicates high DNAm, and blue grey indicates low DNAm. For the heatmap, data are normalized for visualization (mean = 0, variance = 1).
