# Supplementary material for: An Epigenetically Distinct Subset of Children With Autism Spectrum Disorder Resulting From Differences in Blood Cell Composition
Source: Front Neurol. 2021 Apr 16;12:612817. doi: 10.3389/fneur.2021.612817 (PMC8085304; doi:10.3389/fneur.2021.612817)
Supplement: Supplementary file 5 [file Image_4.PDF]

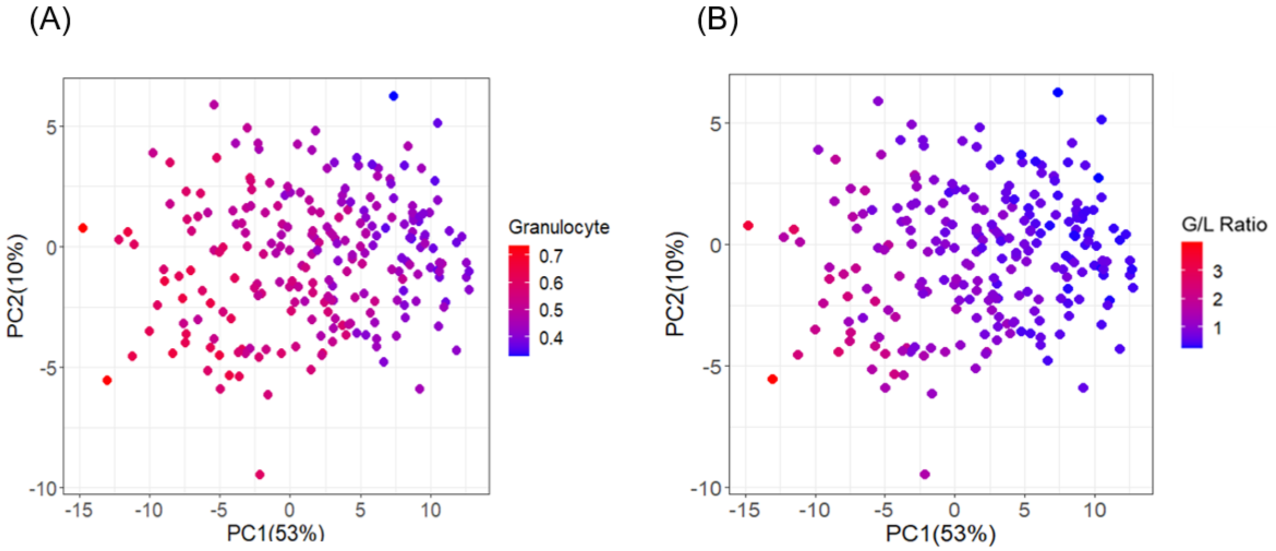

Figure S4. Association between DNAm variation and (**A**) granulocytes proportion and (**B**) granulocyte/lymphocyte (G/L) ratio across ASD cases excluding the 32 ASD cases that were detected as unique both epigenetically and in blood cell composition. Principal component analysis (PCA) plots run on the 77 differentially methylated sites identified between the remaining ASD cases (n = 233), and controls (n=122). The color gradient indicates relative cell type proportion.
