# Supplementary material for: An Epigenetically Distinct Subset of Children With Autism Spectrum Disorder Resulting From Differences in Blood Cell Composition
Source: Front Neurol. 2021 Apr 16;12:612817. doi: 10.3389/fneur.2021.612817 (PMC8085304; doi:10.3389/fneur.2021.612817)
Supplement: Supplementary file 6 [file Image_5.PDF]

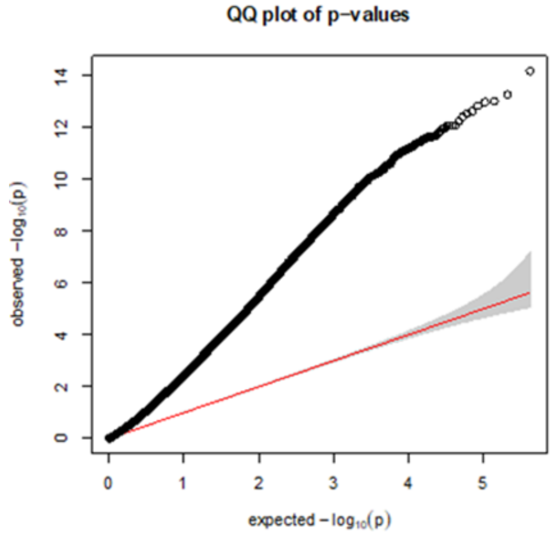

Figure S5. The quantile-quantile plot showing the log-transformed distributions of *limma* regression *p*-values associated with the DNAm differences in ASD cases compared to matching controls. The red line depicts a theoretical line according to the normal distribution.
